# The development and experimental validation of hypoxia-related long noncoding RNAs prognostic signature in predicting prognosis and immunotherapy of cutaneous melanoma

**DOI:** 10.18632/aging.205157

**Published:** 2023-11-02

**Authors:** Gang Wang, Yuliang Sun, Qingjia Xu

**Affiliations:** 1 Department of Orthopedics, Qilu Hospital of Shandong University, Jinan, China

**Keywords:** cutaneous melanoma, hypoxia-related long noncoding RNAs, consensus clustering analysis, immune infiltration landscape, prognosis

## Abstract

Cutaneous melanoma (CM) is widely acknowledged as a highly aggressive form of malignancy that is associated with a considerable degree of morbidity and poor prognosis. Despite this recognition, the precise role of hypoxia-related long noncoding RNAs (HRLs) in the pathogenesis of CM remains an area of active research. This study sought to elucidate the contribution of HRLs in CM by conducting a thorough screening and extraction of hypoxia-related genes (HRGs). In particular, we conducted univariate and multivariate Cox regression analyses to assess the independence of the prognostic signature of HRLs. Our results demonstrated that a novel risk model could be established based on five prognostic HRLs. Remarkably, patients with low-risk scores exhibited significantly higher overall survival rates compared to their high-risk counterparts, as confirmed by Kaplan-Meier survival analysis. Furthermore, we utilized consensus clustering analysis to categorize CM patients into two distinct subtypes, which revealed marked differences in their prognosis and immune infiltration landscapes. Our nomogram results confirmed that the HRLs prognostic signature served as an independent prognostic indicator, offering an accurate evaluation of the survival probability of CM patients. Notably, our findings from ESTIMATE and ssGSEA analyses highlighted significant disparities in the immune infiltration landscape between low- and high-risk groups of CM patients. Additionally, IPS and TIDE results suggested that CM patients in different risk subtypes may exhibit favorable responses to immunotherapy. Enrichment analysis and GSVA results indicated that immune-related signaling pathways may mediate the role of HRLs in CM. Finally, our tumor mutation burden (TMB) results indicated that patients with low-risk scores had a higher TMB status. In summary, the establishment of a risk model based on HRLs in this study provided an accurate prognostic prediction and correlated with the immune infiltration landscape of CM, thereby providing novel insights for the future clinical management of this disease.

## INTRODUCTION

Cutaneous melanoma (CM) is a highly metastatic and aggressive malignancy with an increasing incidence worldwide [1]. Accounting for 5% of skin malignancies and causing more than 70% of deaths, CM poses a significant challenge to human health [2]. The main cause of death in melanoma patients is widespread, and the 5-year survival rate is less than 23% [3]. Despite the promising attention received by immunotherapies such as PD-1/PD-L1 and CTLA4 in CM treatment, the prognosis of CM remains unsatisfactory [4]. Therefore, there is an urgent need to investigate the mechanisms underlying the tumorigenesis and progression of CM to identify novel markers for its diagnosis and treatment.

Hypoxia is the most important and prevalent characteristic of the microenvironment in tumors and is closely associated with tumor proliferation and metastasis, which typically indicates poor prognosis [5]. Several reports have indicated that the majority of malignant tumors are associated with hypoxia, including prostate cancer, glioblastoma multiforme, malignant melanoma, breast cancer, and metastatic liver cancer [6]. In tumor biology, the vasculature of tumors is often unable to keep pace with the rapidly proliferating tumor cells, resulting in highly hypoxic regions. This aggressive phenotype in tumors is further conferred by upregulating angiogenic, survival, proliferative, and metastatic pathways [7]. Previous studies have demonstrated that hypoxia is a well-accepted aggravating factor in tumor development that facilitates metastasis, such as promoting lymph node metastasis in melanoma [8]. It has been reported that hypoxia can promote uveal melanoma cell angiogenesis and metastasis by upregulating the expression of glycosylate - secreted protein ANGPTL4 and VEGF [9]. Notably, as a characteristic feature of virtually all solid tumors, hypoxia can also directly modulate the tumor immune microenvironment [10]. Hypoxia can lead to tumor immunosuppression and immune escape [11]. However, few studies have described the underlying mechanisms of hypoxia in CM.

Long non-coding RNAs (lncRNAs) are a novel class of non-protein-coding transcripts longer than 200 nucleotides that lack apparent protein coding potential [12, 13]. Previous studies have reported that lncRNAs are expressed abnormally in multiple diseases and participate in various biological and physiological processes, including cell proliferation, apoptosis, and migration [14, 15]. With the in-depth study of lncRNAs, dysregulation of lncRNAs has been found to be involved in the tumorigenesis and development of human tumors, including melanoma [16]. Furthermore, lncRNAs have close relationships with the development of CM, including cell cycle arrest, inhibition of tumor microenvironment formation, activation of tumor cell signal pathway, and poor prognosis [17, 18]. Nevertheless, the role of hypoxia-related long noncoding RNAs (HRLs) in CM remains elusive.

Currently, bioinformatics approaches are widely utilized to characterize diseases, and lncRNA-based prognostic models have been developed to evaluate patient prognosis [15]. In this study, an HRL-based model was established using The Cancer Genome Atlas (TCGA) database, and 5 HRL signatures were used to predict the prognosis and evaluate the immune infiltration landscape of CM patients. Moreover, the response to drug sensitivity and immunotherapy of patients in different risk subtypes was comprehensively investigated, and the possible molecular mechanisms were illustrated in detail. Abnormal expressions of HRLs in CM were further verified in cell lines using qRT-PCR. Collectively, the findings of this study provide novel insights and perspectives for the clinical management of CM patients.

## MATERIALS AND METHODS

### TCGA dataset collection

For this study, we obtained the transcriptome matrix in RNA-seq FPKM format and clinical information from The Cancer Genome Atlas database (TCGA) (https://portal.gdc.cancer.gov/). We excluded patients with melanoma who had no survival time or a survival time of less than zero from the analysis, resulting in 454 melanoma samples for further analysis. To annotate the transcriptome matrix symbols (mRNA and lncRNA), we used the ensembles human genome browser GRCh38.p13 (http://asia.ensembl.org/index.html) with the assistance of Perl scripts. We retrieved the patients’ clinical characteristics, such as age, gender, stage, and TN stage, from the TCGA database using Perl scripts. We excluded samples in M stage due to the significant difference in sample size. All clinical information and data were obtained from public databases, and therefore, written informed consent from patients and approval from the ethics committee were not required for this study.

### Identification of HRLs and risk model construction

We obtained 200 hypoxia-related genes (HRGs) from the Molecular Signature Database (MSigDB) (https://www.gsea-msigdb.org/gsea/) (details provided in Supplementary Table 1). Subsequently, we performed Pearson correlation analysis to identify a set of 186 lncRNAs that were significantly associated with HRGs, termed HRLs, based on a threshold of |correlation coefficient| > 0.5 and P < 0.001 (|r| > 0.5, P < 0.001) (details provided in Supplementary Table 2). Using Perl scripts, we extracted the expression levels of the HRLs from the transcriptome matrix and merged them with the corresponding clinical characteristics information for further analysis. We performed univariate Cox regression analysis using the R package “survival” to identify HRLs significantly associated with the overall survival (OS) rate of CM. From this, we selected 51 HRLs that met the predefined criteria (details provided in Supplementary Table 3) for further analysis. The least absolute shrinkage and selection operator (LASSO) regression analysis was employed using the R package “glmnet” to identify the most characteristic variables among the prognostic HRLs. The candidate HRLs were selected using multivariate Cox regression analysis, which was performed using the R package “survival” to establish the risk model. The risk score for each CM patient was calculated using the following formula: risk score = (-0.357 x expression of LINC00324) + (0.221 x expression of EBLN3P) + (0.218 x expression of MIR205HG) + (-0.118 x expression of THCAT158) + (-0.439 x expression of USP30-AS1). Based on the median risk score, patients were divided into low- and high-risk groups. We used the Kaplan-Meier survival curve to evaluate the OS rate of patients in the two risk groups using the log-rank algorithm with the R package “survival”. We used the R package “ggplot2” to perform principal component analysis (PCA) to investigate the distribution pattern between the low- and high-risk groups. We visualized the expression of HRLs in the low- and high-risk groups using the R package “pheatmap”.

### Function enrichment analysis and tumor mutational burden landscape

The tumor mutation data in maf format of CM samples were retrieved from the TCGA database. Perl scripts were used to extract the mutation data from the raw data, and the “Maftools” package in R software was employed to create a waterfall diagram. To identify differentially expressed genes (DEGs) between patients in the low- and high-risk group, the R package “limma” was utilized, with a threshold of |fold change| ≥ 2 and P < 0.05. The KEGG terms of CM patients in the low- and high-risk group were calculated using Gene Set Variation Analysis (GSVA) with P < 0.05 considered significantly different. Furthermore, the “clusterProfiler” R package was used to conduct Gene Ontology (GO) and Kyoto Encyclopedia of Genes and Genomes (KEGG) analysis to enrich the DEGs into pathways [19].

### Independent prognosis analysis

Univariate and multivariate Cox regression analyses are commonly used statistical methods to examine the association between survival outcomes and various factors, such as the HRLs prognostic signature and clinicopathological characteristics. The pROC package is utilized to assess the accuracy of diagnostic tests and predict AUC values. The timeROC package is employed to compute the AUC values for different time points. Nomogram models can integrate diverse prognostic factors to predict the survival probability, and the rms package is used to construct nomograms. The C-index plot is a tool to evaluate the predictive accuracy of survival models. The regplot package can be used to generate calibration diagrams that compare the predicted survival probability with the actual survival probability. The ggDCA package is employed to perform decision curve analysis, which assesses the clinical utility of prediction models.

### Validation of risk model and consensus clustering analysis

The CM samples were divided into training and test cohorts in a random manner using the R package “caret,” at a ratio of 7:3. A total of 318 CM samples were assigned to the training cohort, while 136 samples were assigned to the test cohort. Utilizing the prognostic HRLs, the risk score for each CM sample was calculated according to the risk formula in both cohorts. The patients in both cohorts were subsequently categorized into low- and high-risk groups based on the median risk score. To classify the CM samples into different molecular subtypes, the R package “ConsensusClusterPlus” was employed. The clustering was based on partitioning around medoids with “euclidean” distances, using 1000 iterations, and with the maximum K value set at 9. Based on the optimal classification of K between 2 and 9, the CM samples were assigned to different molecular subtypes for further analysis.

### Cell culture and qRT-PCR analysis

The human fibroblasts cell line HFB4 and human melanoma cell line A375 were obtained from the American Type Culture Collection (ATCC). To initiate the culture, the cryopreserved A375 and HFB4 cells were thawed in a 37° C water bath and transferred to sterile 15 mL centrifuge tubes containing 10 mL of DMEM/F12 culture medium under aseptic conditions. The tubes were then incubated in a humidified incubator at 37° C with 5% CO2 to promote cell growth and maintenance of cell viability. Subsequently, RNA extraction from both cell lines was performed using Trizol reagent (Catalogue number: 15596018, Thermo Fisher Scientific), followed by cDNA synthesis using a reverse transcription kit with gDNA Eraser (Perfect Real Time, Takara Bio). Real-time quantitative qRT-PCR (Catalogue number: RR047A, Takara Bio) was carried out to perform further analysis. Finally, the lncRNA expression levels were measured using SYBR Pre-mix Ex Taq II (TliRNaseH Plus) (Catalogue number: RR820B, Takara Bio).

### Immune infiltration landscape, immunotherapy response and drug sensitivity analysis

ESTIMATE algorithm was used to estimate the proportion of stromal and immune cells in CM samples, and the stromal, immune, ESTIMATE scores, and tumor purity were estimated using R package “estimate”. Then, single sample gene set enrichment analysis (ssGSEA) algorithm was used to evaluate the proportion of 23 types of immune cells and the immune function score of each CM sample via the “GSVA” R package. Spearman-ranked correlation analysis was used to investigate the association of prognostic IHRLs and immune cells, which were visualized in a heatmap using the “ggplot2” R package. In addition, the Immunophenoscore (IPS) results of CM patients were downloaded from the TCIA database, and the TIDE score of each sample was calculated using the TIDE database. Finally, the Genomics of Drug Sensitivity in Cancer (GDSC) database was utilized to evaluate drug sensitivity (IC50), and the response to antineoplastic drugs for each CM sample was predicted using the “pRRophetic” R package. Correlation analysis was used to investigate the correlation between risk score and drug sensitivity (IC50), and all statistical analysis were visualized using the “ggplot2” R package.

### Statistical analysis

In this study, all statistical analyses were conducted using R software version 4.1.0 (http://www.R-project.org) and Perl scripts. The correlation between two variables was calculated using the Spearman’s rank correlation algorithm, and statistical significance was set at a threshold of P < 0.05. Differential functions were analyzed using the Wilcoxon rank-sum test between the two groups, and statistical significance was set at a threshold of *p* < 0.05.

### Availability of data and materials

The data used to support the findings of this study are included within the article. The data and materials in the current study are available from the corresponding author on reasonable request.

## RESULTS

### Risk model development based on HRLs prognostic signature in CM

**Figure 1:**
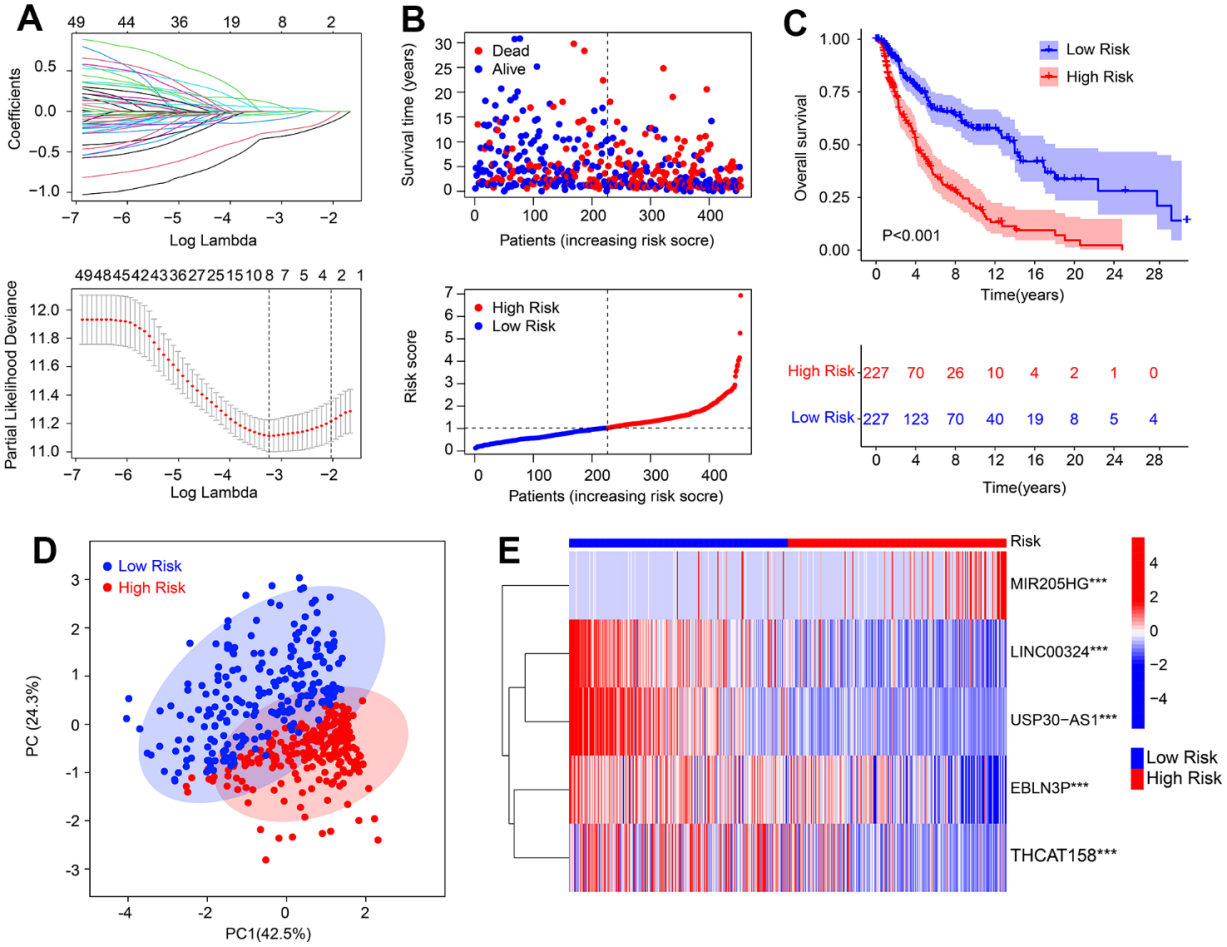
**Risk model construction based on the HRLs prognostic signature in CM.** (**A**) LASSO regression analysis shows the optimal coefficients and minimum lambda. (**B**) Risk score distribution, and the scatter dot plot shows the correlation between risk score and survival time in CM. (**C**) Kaplan-Meier survival curve analysis suggests that the OS rate of patients with low-risk score is longer than those with high-risk score. (**D**) Principal component analysis illustrates a significant separation between low- and high-risk group. (**E**) Heatmap diagram shows the expression of 5 prognostic HRLs of patients in the low- and high-risk group.

In this study, we developed a novel risk model to investigate the prognostic value of HRLs in predicting the prognosis for CM. To identify the HRLs, we performed Pearson correlation analysis and identified 186 lncRNAs that were associated with HRGs (Supplementary Figure 1A). Then, we used the least absolute shrinkage and selection operator (LASSO) analysis and identified 8 HRLs that were associated with OS rate (Figure 1A and Supplementary Figure 1B) based on univariate Cox regression analysis. Multivariate Cox regression analysis showed that 5 HRLs could independently predict the OS rate of CM, and they were used to construct the risk model. Based on the median of risk score, we ranked and divided the patients with CM into low- and high-risk groups. The scatter dot plot showed that the HRLs prognostic signature was inversely associated with survival time in CM (Figure 1B). The Kaplan-Meier survival curve analysis indicated that patients with low-risk scores had a significantly higher OS rate compared to those with high-risk scores (Figure 1C). Moreover, principal component analysis (PCA) illustrated a clear separation between the low- and high-risk groups based on the HRLs prognostic signature (Figure 1D). The heatmap visualizable diagram result showed that the expression of LINC00324, USP30-AS1, EBLN3P, and THCAT158 were significantly higher in the low-risk group, whereas the expression of MIR205HG was higher in the high-risk group (Figure 1E). These findings indicate that the construction of the risk model for the HRLs prognostic signature is closely associated with the prognosis of patients with CM.

### Validation of risk model based on HRLs prognostic signature

**Figure 2:**
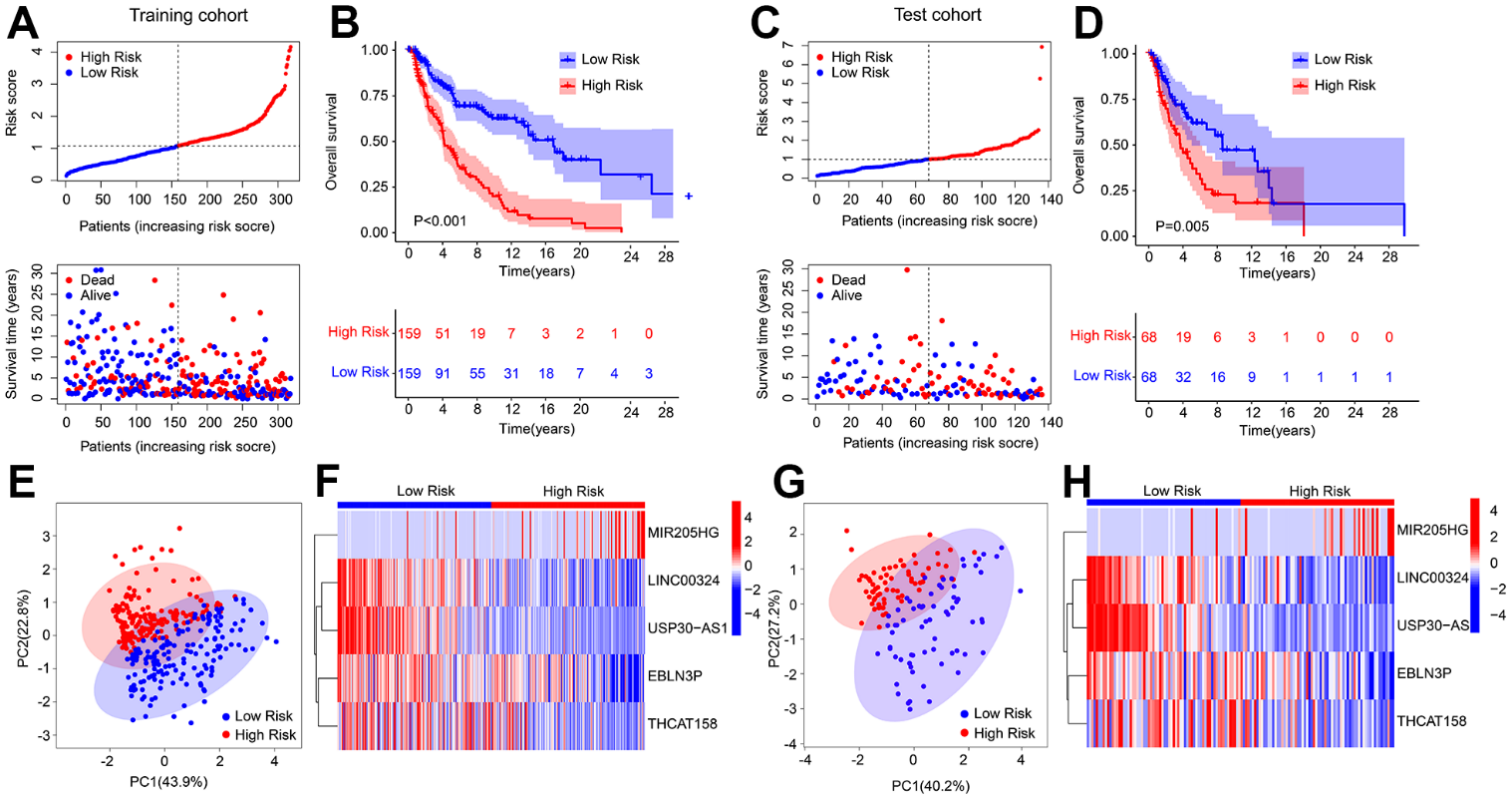
**Risk model construction in training cohort and test cohort based on the prognostic HRLs.** (**A**) Distribution of risk score in training cohort. (**B**) Kaplan-Meier survival curve analysis of patients with CM in training cohort. (**C**) Distribution of risk score in test cohort. (**D**) Kaplan-Meier survival curve analysis of patients with CM in test cohort. (**E**) PCA score plot shows a clear separation between low- and high-risk group in training cohort. (**F**) Heatmap diagram displays the expression of 5 prognostic HRLs in training cohort. (**G**) PCA score plot shows a clear separation between low- and high-risk group in test cohort. (**H**) Heatmap diagram displays the expression of 5 prognostic HRLs in test cohort.

An internal validation was conducted to assess the accuracy and independence of the HRLs prognostic signature in predicting the prognosis of CM patients. The patients were randomly split into a training cohort and a test cohort in a 7:3 ratio, resulting in 318 samples in the training cohort and 136 samples in the test cohort. Using the 5 prognostic HRLs, the risk score of each CM sample was calculated and categorized into low- and high-risk groups in both cohorts. The patients in the training cohort were ranked based on their median risk score, and the scatter dot plot showed an inverse correlation between the risk score and survival time (Figure 2A). Similarly, the patients in the test cohort were ranked according to the median risk score, and the scatter dot plot indicated an inverse correlation between the risk score and survival time (Figure 2C). The Kaplan-Meier survival curve analysis showed that patients in the low-risk group had a significantly higher OS rate than those in the high-risk group (Figure 2D). The PCA score plot revealed a clear separation between the low- and high-risk groups in both cohorts (Figure 2E–2H). Furthermore, the heatmap diagram showed that the low-risk group had higher expression levels of LINC00324, USP30-AS1, EBLN3P, and THCAT158, while the high-risk group had a higher expression level of MIR205HG, in both cohorts. These findings suggest that the HRLs prognostic signature accurately predicts the prognosis of CM patients.

### The prognostic signature based on HRLs was an independent prognosis indicator for CM

**Figure 3:**
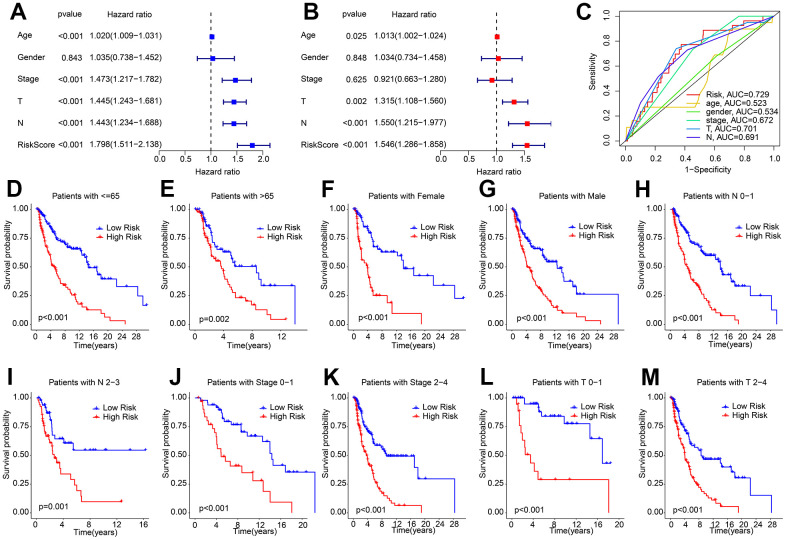
**Independent prognosis analysis of HRLs prognostic signature and clinicopathological characteristics.** (**A**) Univariate Cox regression analysis shows that age, stage, T, N, and risk score are closely associated with OS rate in CM. (**B**) Multivariate Cox regression analysis reveals that risk score is an independent prognostic indicator of patients with CM. (**C**) ROC curve shows the AUC of HRLs prognostic signature and different clinicopathological characteristics. (**D**–**M**) The Kaplan-Meier survival curve shows the OS rate of patients with low- and high-risk score in different clinicopathological characteristics.

Univariate and multivariate Cox regression analyses were employed to assess the ability of the risk score based on HRLs prognostic signature to serve as an independent prognostic indicator for CM. The results of the univariate analysis showed a close association between age (HR = 1.020, P < 0.001), stage (HR = 1.473, P < 0.001), T (HR = 1.445, P < 0.001), N (HR = 1.443, P < 0.001), risk score (HR = 1.798, P < 0.001) and OS rate in patients with CM (Figure 3A). On the other hand, the multivariate Cox regression analysis indicated that age (HR = 1.013, P = 0.025), T (HR = 1.315, P = 0.002), N (HR = 1.550, P < 0.001), and risk score (HR = 1.546, P < 0.001) were all independent prognostic indicators for CM patients (Figure 3B). The ROC curve demonstrated that the risk model had an AUC of 0.729, indicating a satisfactory predictive ability for CM (Figure 3C). Furthermore, a stratified subgroup analysis was conducted to evaluate the prognostic value of the HRLs prognostic signature in different clinicopathological characteristics. The patients with CM were categorized into low- and high-risk groups based on the median risk score, and the analysis was performed across various clinicopathological characteristics, including age (age ≤ 65 vs age > 65), gender (female vs male), N (N 0-1- vs N 2-3), stage (stage 0-1 vs stage 2-4), T (T 0-1 vs T 2-4). The Kaplan-Meier survival curve analysis revealed that the OS rate of patients with a low-risk score was significantly higher than those with a high-risk score across different clinicopathological characteristics (Figure 3D–3M). These results collectively demonstrate that the risk score based on the HRLs prognostic signature is an independent prognostic indicator that can effectively predict the prognosis of CM patients in comparison to other clinicopathological characteristics.

### Nomogram construction based on the HRLs prognostic signature and clinicopathological characteristics

**Figure 4:**
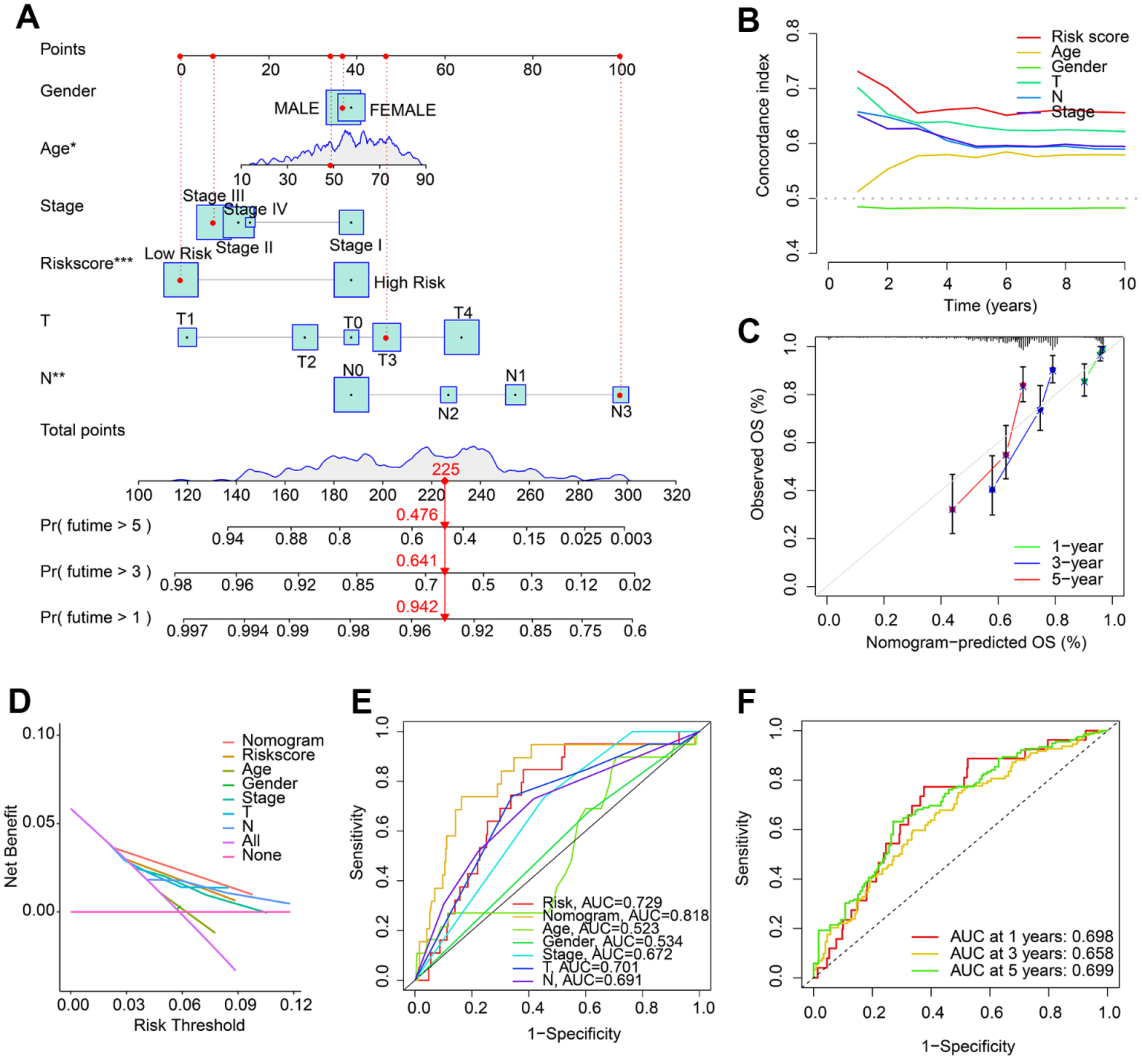
**Nomogram construction based on HRLs prognostic signature and clinicopathological characteristics.** (**A**) Construction of nomogram model to predict 1-, 3-, and 5-years survival probability of patients with CM. (**B**) Concordance index curve of risk score and clinicopathological characteristics. (**C**) Calibration curve reveals the consistence of nomogram-predict OS and actual OS. (**D**) Decision curve analysis (DCA). (**E**) ROC curve shows the AUC of nomogram, risk score, and clinicopathological characteristics. (**F**) Time-dependent ROC curve shows the AUC of 1-, 3-, and 5-years.

A nomogram has been developed to accurately assess the probability of survival at 1, 3, and 5 years for patients with CM, based on their HRLs prognostic signature and clinicopathological characteristics (Figure 4A). The concordance index (C-index) curve showed that the predictive capability of the HRLs signature in predicting prognosis of CM patients was better than other clinicopathological characteristics (Figure 4B). The calibration curve indicated that the predicted OS rates for 1, 3, and 5 years using the nomogram were consistent with the actual OS rates (Figure 4C). The decision curve analysis (DCA) and ROC curve results also suggested a satisfactory predictive ability of the nomogram for predicting the survival probability of CM patients (Figure 4D, 4E). The time-dependent ROC curve indicated that the AUC for 1, 3, and 5 years was 0.689, 0.658, and 0.699, respectively (Figure 4F). In summary, these findings demonstrate that the nomogram construction based on HRLs prognostic signature accurately predicts the prognosis of CM patients and is highly reliable.

### Functional enrichment analysis of differential expressed genes (DEGs)

**Figure 5:**
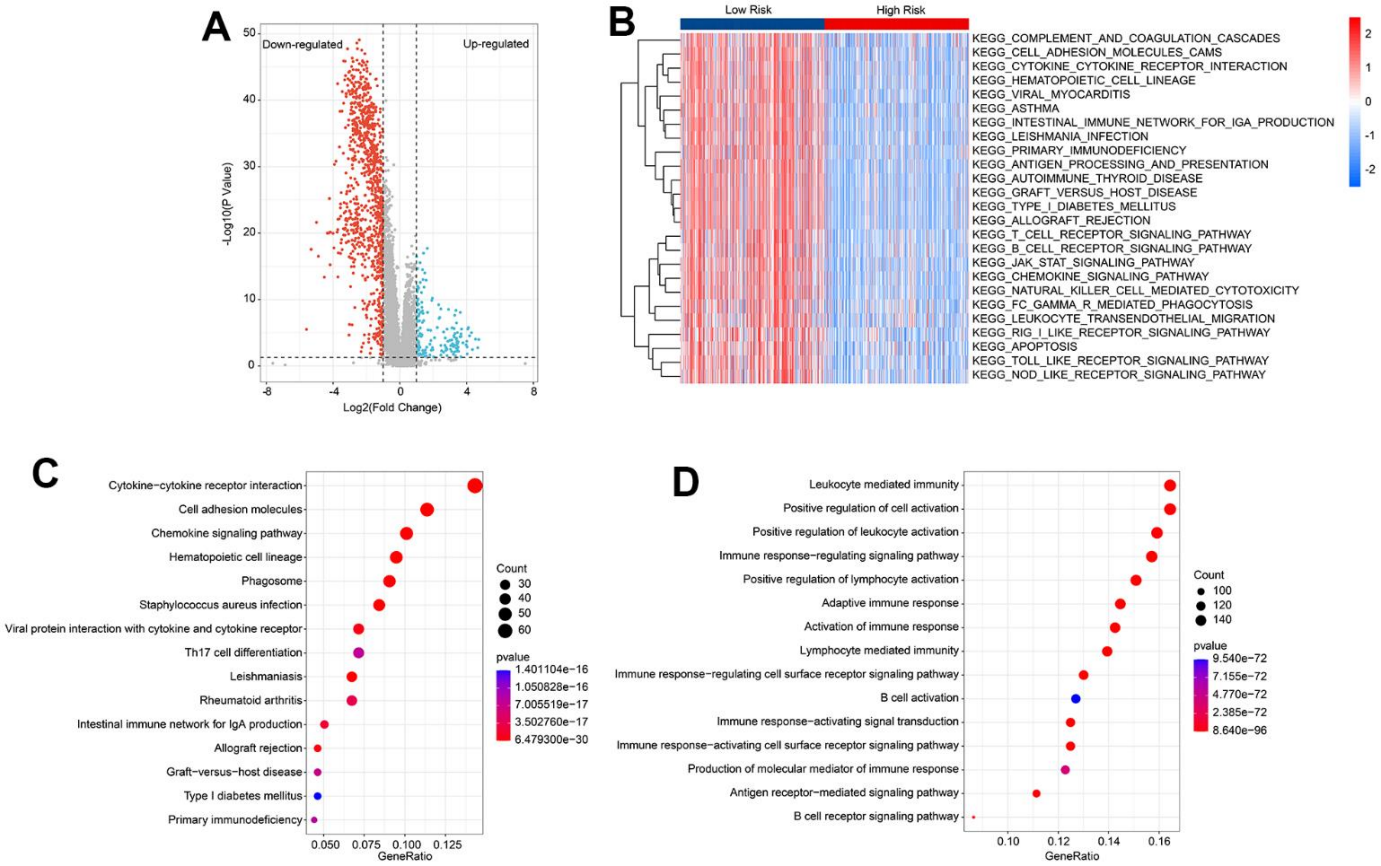
**Functional enrichment analysis of differential expression genes (DEGs) in low- and high-risk group.** (**A**) Volcano diagram shows the DEGs in the low- and high-risk group with the threshold set at |Fold Change| ≥ 2 and P < 0.05. (**B**) GSVA reveals the activity of KEGG signal pathways of each CM patient in the low- and high-risk group. (**C**) KEGG enrichment analysis of DEGs. (**D**) GO enrichment analysis of DEGs.

To investigate the potential molecular mechanisms of CM patients in the low- and high-risk groups, GSVA and enrichment analysis were employed. The volcano diagram (Figure 5A) illustrates the differentially expressed genes (DEGs) in the low- and high-risk groups. GSVA was used to calculate the activity of KEGG pathways, and the results suggested a remarkable down-regulation in immune-related signaling pathways of CM patients in the high-risk group (Figure 5B). KEGG enrichment analysis indicated that the DEGs were significantly enriched in cytokine-cytokine receptor interaction, cell adhesion molecules, and chemokine signaling pathways (Figure 5C). GO enrichment analysis illustrated that DEGs were enriched in immune-related biological processes, such as leukocyte-mediated immunity, positive regulation of cell activation, and positive regulation of leukocyte activation (Figure 5D). These results suggest that immune-related processes may play a role in mediating the effects of HRLs in CM patients.

### Consensus clustering analysis and immune infiltration landscape

**Figure 6:**
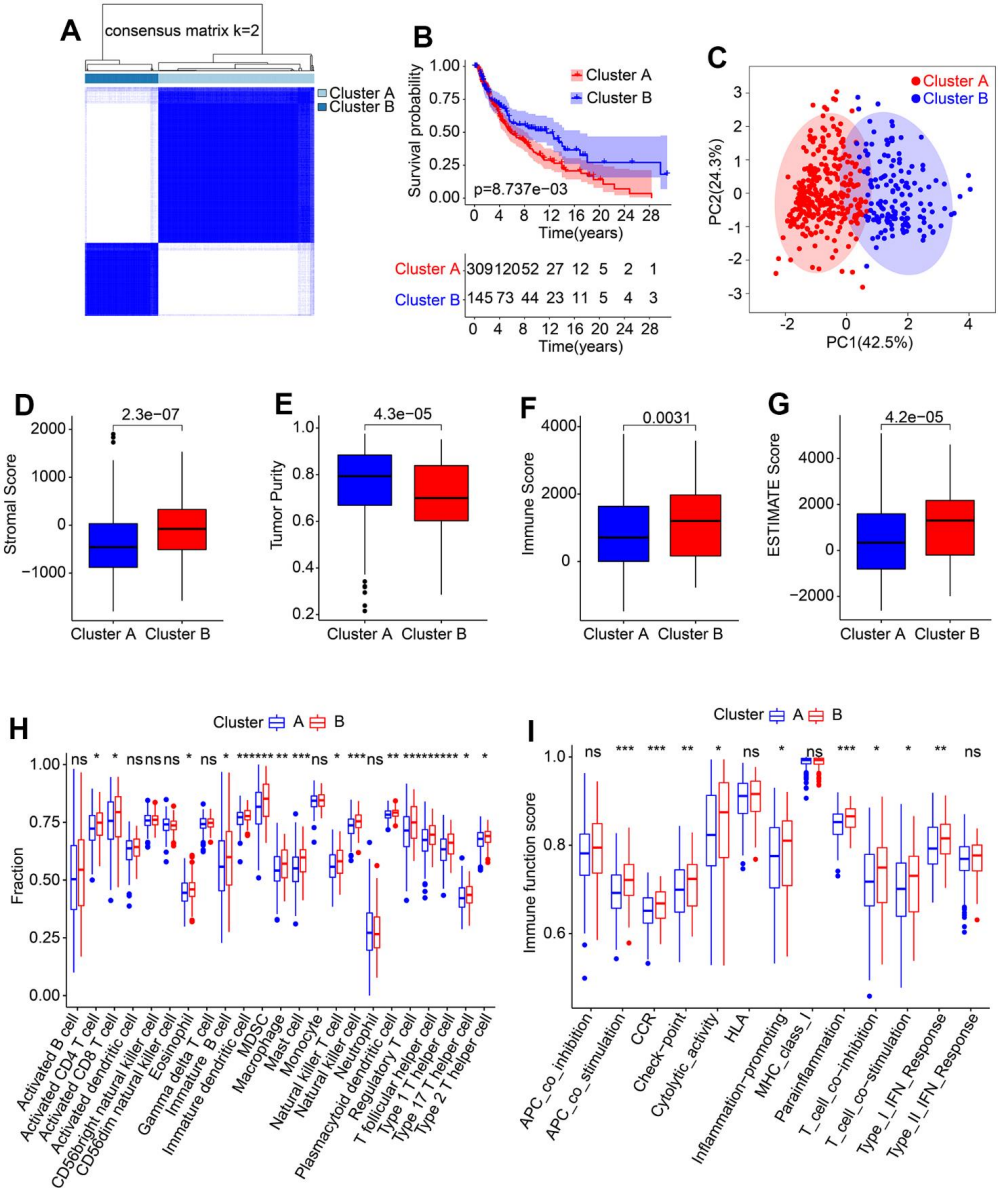
**Consensus clustering of CM samples and immune infiltration landscape evaluation.** (**A**) Consensus clustering heatmap shows the optimal classification under K= 2-9. (**B**) The Kaplan-Meier survival curve analysis of patients with CM in Cluster A and Cluster B. (**C**) PCA score plot illustrates a clear distribution between Cluster A and Cluster B. (**D**–**G**) Stromal, immune and ESTIMATE scores, and tumor purity. (**H**) The proportion of 23-type immune cells in Cluster A and Cluster B. (**I**) Immune function score of patients in Cluster A and Cluster B.

The molecular subtypes of cutaneous melanoma (CM) were further investigated using a set of five prognostic HRLs. Consensus clustering was employed to classify the CM patients into different molecular subtypes, resulting in an optimal classification of K=2, with 309 samples in Cluster A and 145 samples in Cluster B, as illustrated in the heatmap (Figure 6A). Kaplan-Meier survival analysis revealed a significant difference in the overall survival (OS) rate between patients in Cluster A and Cluster B, with Cluster A exhibiting a higher OS rate than Cluster B (Figure 6B). Additionally, the results of principal component analysis (PCA) demonstrated a clear separation of patients in Cluster A and Cluster B based on the five prognostic HRLs (Figure 6C). Furthermore, the ESTIMATE assessment algorithm indicated that patients in Cluster B had higher stromal, immune, and ESTIMATE scores and lower tumor purity than those in Cluster A (Figure 6D–6G). Similarly, the results of the ssGSEA algorithm revealed that the proportion of most immune cells was higher in patients in Cluster B (Figure 6H). Moreover, immune function analysis demonstrated that patients in Cluster B had a higher immune function score than those in Cluster A, as evidenced by higher cytolytic activity and T cell co-inhibition (Figure 6I). These findings demonstrate that the five prognostic HRLs can accurately classify CM samples into different molecular subtypes that are associated with prognosis and immune infiltration landscape.

### Association of HRLs prognostic signature and immune infiltration landscape

**Figure 7:**
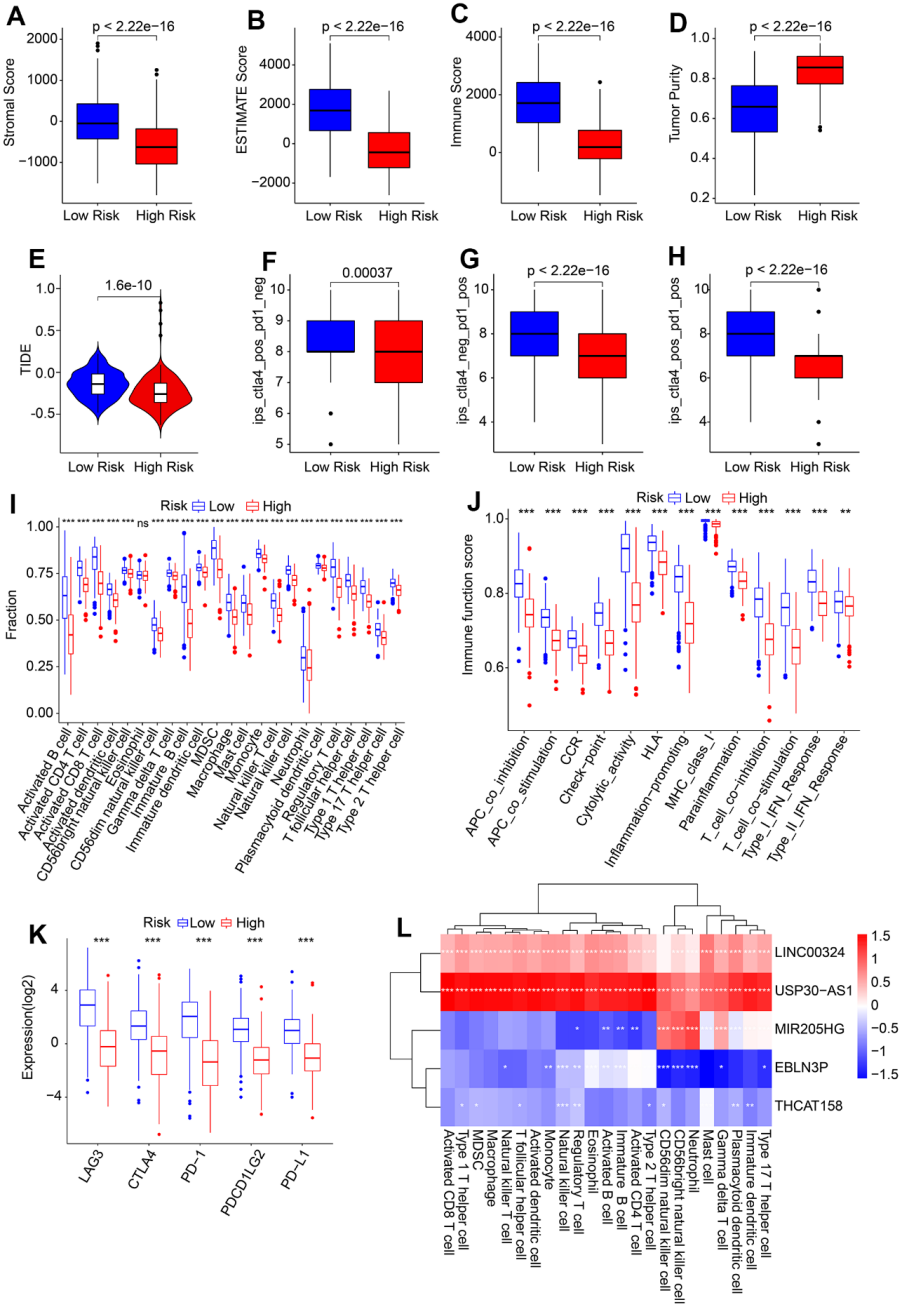
**Association of risk score and immune infiltration landscape in CM.** (**A**–**D**) Stromal, immune, ESTIMATE scores, and tumor purity. (**E**) TIDE score. (**F**–**H**) IPS score. (**I**) The proportion of 23-type immune cells of patients in the low- and high-risk group. (**J**) Immune function score. (**K**) Expression of immune checkpoints inhibitor (ICI) of in low- and high-risk group. The expression of ICI was transformed by log2 (expression + 1). (**L**) Correlation analysis of 5 prognostic HRLs and 23-type immune cells.

The present study investigated the association between the HRLs prognostic signature and the immune infiltration landscape. The ESTIMATE analysis showed that patients in the low-risk group had higher stromal, immune, and ESTIMATE scores, and lower tumor purity (Figure 7A–7D). Additionally, the immunotherapy prediction analysis indicated that patients with high-risk scores had lower TIDE scores, suggesting a better response to immunotherapy for patients in the high-risk group (Figure 7E). Furthermore, the response to anti-CTLA4 and anti-PD-1 immunotherapies was evaluated for CM patients in the low- and high-risk groups. The IPS analysis suggested that the low-risk group showed a promising response to anti-CTLA4, antiPD-1, and anti-CTLA4/PD-1 (Figure 7F–7H). ssGSEA and immune function score analysis revealed that patients in the low-risk group had a higher proportion of immune cells and immune function scores than those in the high-risk group (Figure 7I, 7J). Moreover, the expression of ICI analysis showed higher expression of LAG3, CTLA4, PD−1, PDCD1LG2, and PD−L1 in the low-risk group (Figure 7K). Finally, a correlation analysis was conducted to investigate the association between the 5 prognostic HRLs and immune infiltration landscape. The results showed that LINC00324 and USP30−AS1 were positively correlated with 23 types of immune cells, while EBLN3P and THCAT158 were negatively associated with most of the 23 types of immune cells (Figure 7L). These findings suggest that the risk model based on HRLs prognostic signature is closely associated with the immune infiltration landscape and may aid in the evaluation of the immunotherapy response of CM patients in different risk subgroups.

### Correlation analysis of risk score and drug sensitivity

**Figure 8:**
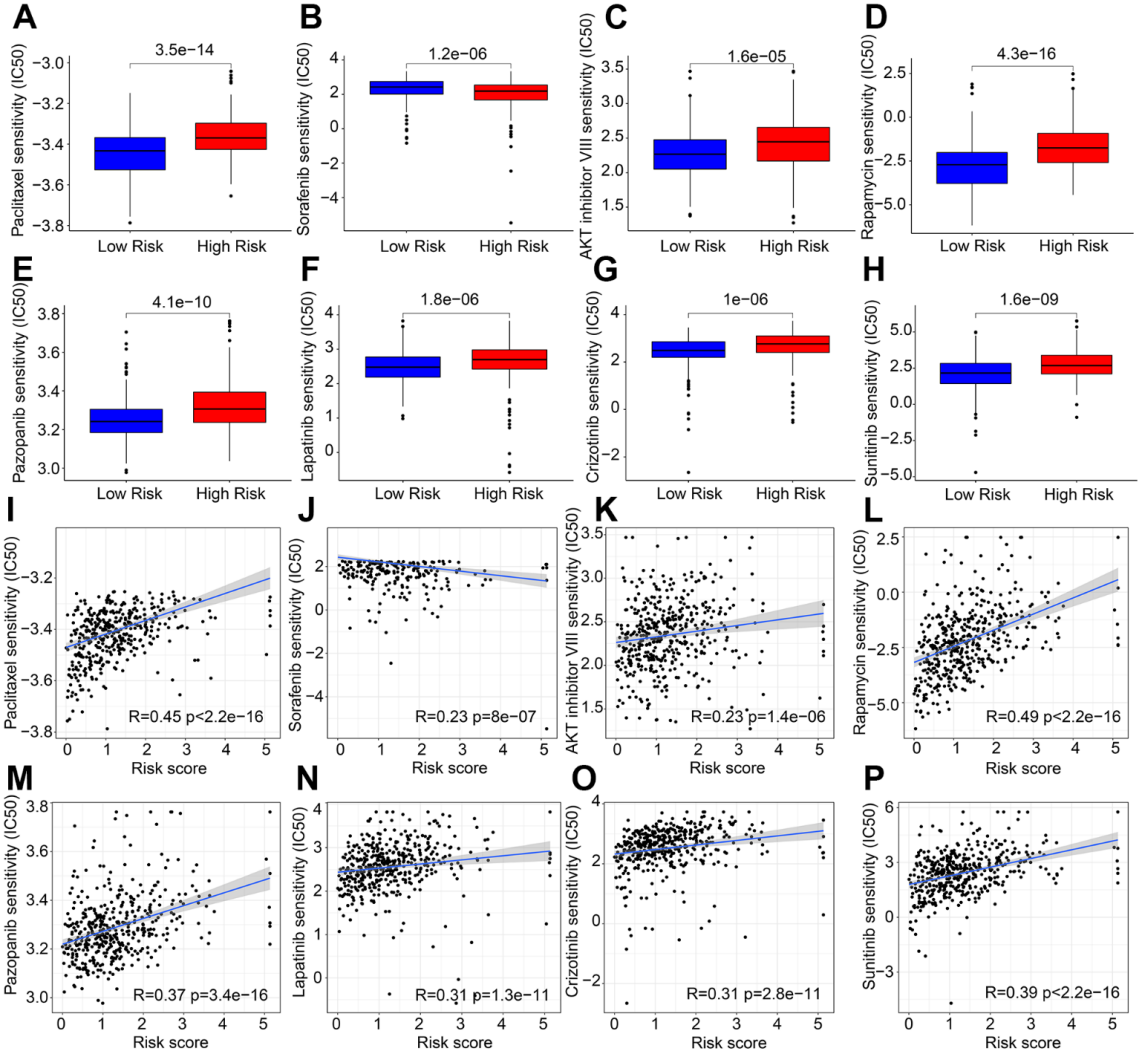
**Correlation analysis of risk score and drug sensitivity.** IC 50 of (**A**) Paclitaxel, (**B**) Sorafenib, (**C**) AKT inhibitor VIII, (**D**) Rapamycin, (**E**) Pazopanib, (**F**) Lapatinib, (**G**) Crizotinib, and (**H**) Sunitinib. (**I**–**P**) Correlation analysis of risk score and frug sensitivity.

Targeted drug therapy has emerged as a promising approach in treating patients with cutaneous melanoma (CM). In light of the substantial differences in immunotherapy responses among CM patients in various risk subgroups, we conducted further investigations to assess the sensitivity of antineoplastic drugs in different risk groups. Our results, as depicted in Figure 8A–8H, demonstrate that the IC 50 values of Paclitaxel, AKT inhibitor VIII, Rapamycin, Pazopanib, Lapatinib, Crizotinib, and Sunitinib were significantly higher in the high-risk group, whereas the IC 50 of Sorafenib was significantly higher in the low-risk group. Furthermore, correlation analysis revealed a positive correlation between risk score and Paclitaxel, AKT inhibitor VIII, Rapamycin, Pazopanib, Lapatinib, Crizotinib, and Sunitinib, but a negative correlation with Sorafenib (Figure 8I–8P). Taken together, these results suggest that CM patients in different risk subgroups may exhibit distinct sensitivities to antineoplastic drugs, providing a new perspective for the development of precisely targeted drug and chemotherapy for CM.

### The landscape of somatic gene mutations based on the HRLs prognostic signature

**Figure 9:**
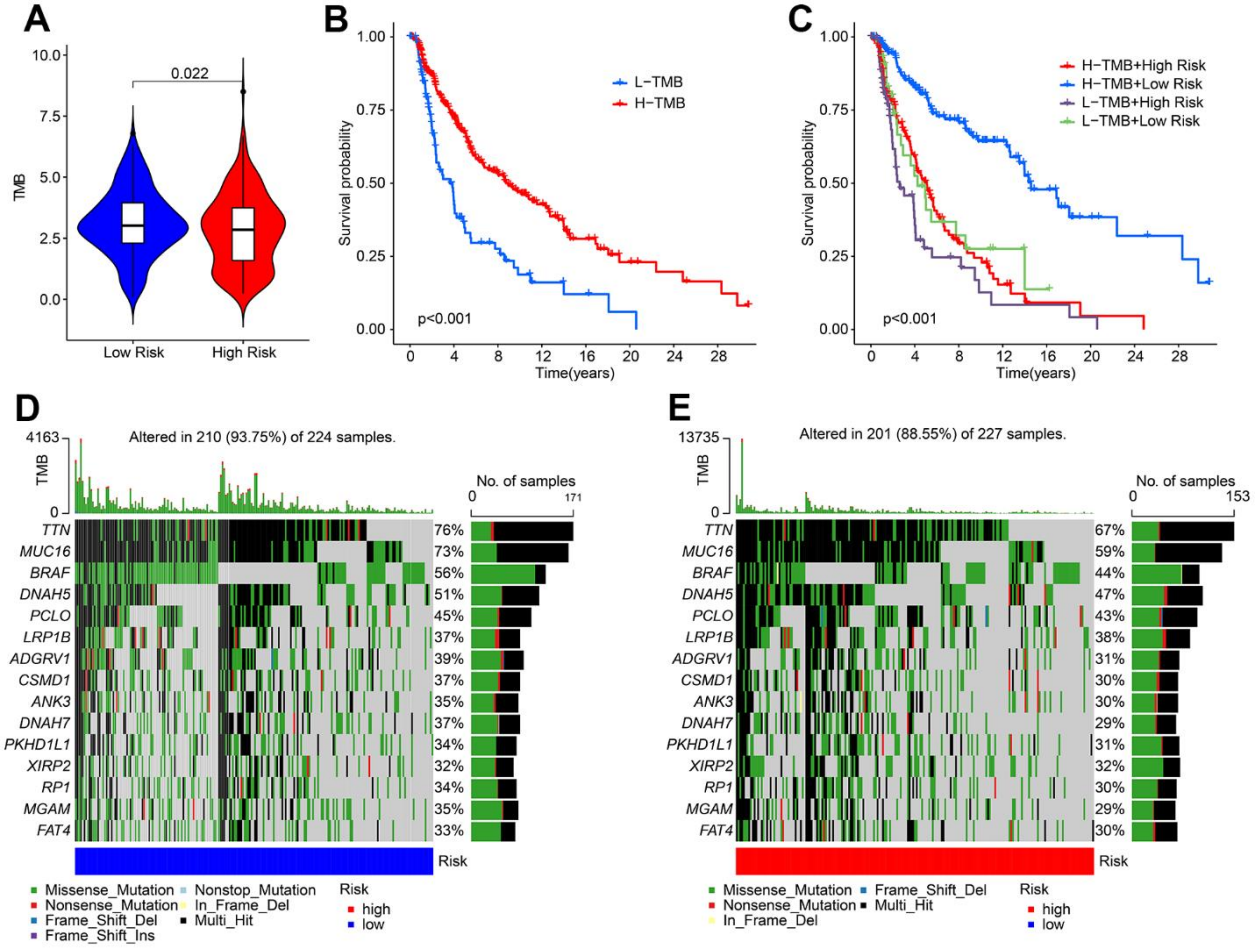
**The tumor mutational burden landscape of patient in the low- and high-risk group.** (**A**) TMB analysis in the low- and high-risk group. (**B**) The Kaplan-Meier survival curve analysis of patients with low- and high-TMB. (**C**) Kaplan-Meier survival curve of patient with L-TMB and H-TMB in the low- and high-risk group. (**D**, **E**) The mutant landscape of CM patients in the low- and high-risk group.

Tumor mutational burden (TMB) has emerged as a promising biomarker for predicting immunotherapy response in tumors. Our analysis of TMB indicated that patients in the low-risk group exhibited a higher TMB compared to those in the high-risk group (Figure 9A). Furthermore, Kaplan-Meier survival curve analysis revealed that patients with a high-risk score had a significantly lower overall survival (OS) rate than those with a low-risk score (Figure 9B). Additionally, the OS rate of patients with a low-risk score was significantly higher than those with a high-risk score in both H-TMB and L-TMB groups (Figure 9C). Analysis of the mutation frequencies of genes revealed that the low-risk group had higher mutation frequencies in most genes, including TTN, MUC16, BRAF, DNAH5, and PCLD (Figure 9D, 9E). These findings suggest that TMB may be a useful biomarker for predicting immunotherapy response in CM patients, and that patients in the low-risk group may benefit from immunotherapy treatment.

### *In vitro* validation of the expression levels of five independent prognostic factors

**Figure 10:**
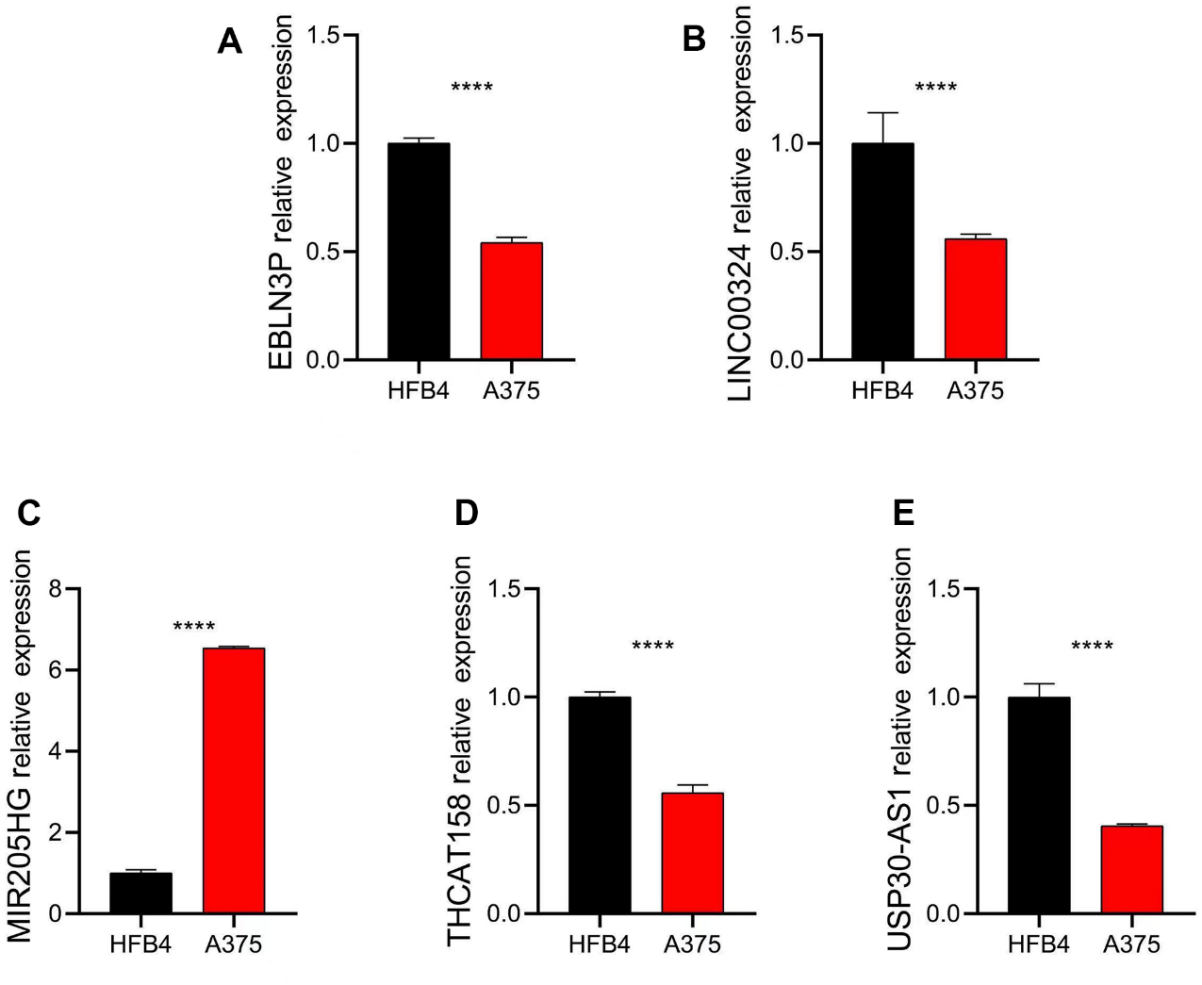
**Relative expression of five independent prognostic factors in HFB4 and A375 cell lines.** Relative expression of (**A**) EBLN3P; (**B**) LINC00324; (**C**) MIR205HG; (**D**) THCAT158; (**E**) USP30-AS1. Data: mean±SD, *P<0.05, **P<0.01, ***P<0.001, ****P<0.0001.

To validate the results of the public database, we assessed the expression of five distinct prognostic factors in both normal and CM cells using the human fibroblasts cell line HFB4 and human melanoma cell line A375. Our findings indicate that EBLN3P, LINC00324, THCAT158, and USP30-AS1 were significantly overexpressed in HFB4 cells, while MIR205HG was significantly overexpressed in A375 cells (Figure 10).

## DISCUSSION

CM is a malignant skin tumor with a poor prognosis, and its incidence has been increasing in recent years. CM has the ability to metastasize and evade immune and cytotoxic attacks, making conventional therapies, particularly chemotherapy, insufficient [16, 20]. While early-stage CM is usually treated with surgical resection, immunotherapy has shown positive outcomes for inoperable or metastatic cases [21, 22]. Notably, the mechanisms involved in immunotherapy are too broad and individual differences in different patients, and the prognosis of cancer patients through immunotherapy also varies from different population [18, 23]. Thus, exploring CM in-depth and discovering new biomarkers and diagnostic options are critical for improving patient outcomes.

The tumor microenvironment, particularly the hypoxic milieu, is known to play a significant role in the regulation of tumor cells, thereby influencing tumor progression and response to antitumor therapy [24, 25]. A mounting body of evidence indicates that the hypoxic microenvironment exerts a profound impact on tumor progression and response to therapy [26, 27]. The hypoxic microenvironment can facilitate tumor progression, metastasis, and heterogeneity, while also eliciting a range of immunosuppressive responses, such as modulating the levels of macrophages, natural killer cells, and T cells [28, 29]. Long non-coding RNAs (lncRNAs) are recognized to play a pivotal role in the communication between cancer cells and the hypoxic microenvironment.

Long non-coding RNAs (lncRNAs) have emerged as critical players in various aspects of tumorigenesis, particularly as novel biomarkers for tumor diagnosis and prognosis [30]. However, current research on the relationship between lncRNAs, hypoxia, and CM mainly focuses on individual or select lncRNAs, and a systematic study on the prognostic prediction of CM patients using high-risk lncRNA (HRL) signatures is lacking. Therefore, it is crucial to establish HRL signatures based on large-scale databases for accurate prognosis prediction in CM. In this study, we developed a novel risk model based on five HRLs. Univariate and multivariate Cox regression analyses revealed that the risk score derived from the HRL prognostic signature could serve as an independent prognostic factor, distinguishing it from other clinicopathological characteristics. Functional enrichment analysis demonstrated that HRLs may be involved in immune-related processes in CM. Results from immune infiltration analysis revealed that the HRL-based risk model could reflect the immune status of CM patients, providing insights for individualized immunotherapy. Additionally, drug sensitivity analysis revealed differences in response to antineoplastic drugs between the two CM subgroups identified by our risk model. Furthermore, mutation frequency analysis indicated that the low-risk group had higher mutation frequencies. Collectively, our findings provide a comprehensive view for the treatment of CM, contributing to the improvement of patient prognosis.

Recent evidence suggests that long non-coding RNAs (lncRNAs) play a significant role in tumor tumorigenesis, invasion, and metastasis, and their dysregulated expression is associated with the progression and recurrence of various cancers, including cutaneous melanoma (CM). In our study, we found that the expression levels of LINC00324, USP30-AS1, EBLN3P, and THCAT158 were significantly higher in the low-risk group, whereas the high-risk group exhibited higher expression of MIR205HG. LINC00324, a 2115 bp lncRNA located on chromosome 17p13.1, has been found to be abnormally expressed in multiple human cancer types, correlating with tumor initiation and progression [31, 32]. Ding et al. reported that LINC00324 is a protective factor in melanoma patients [33], which is consistent with our findings. USP30-AS1, transcribed from the antisense strand of the USP30 gene, has been implicated in autophagy and is a potential prognostic indicator in cancer, according to Sun et al. [34].

Chen et al. revealed that USP30-AS1 significantly inhibited cell proliferation, migration, and invasion *in vitro*, as well as tumor growth *in vivo* [35]. EBLN3P (Endogenous bornavirus-like nucleoprotein) is a recently identified lncRNA located on the forward strand of chromosome 9:37,079,935–37,086,874 [36]. EBLN3P has been found to be prognostic and incorporated into prognostic lncRNA signatures in multiple tumors [37, 38]. Clinical assays have demonstrated that high EBLN3P expression is positively correlated with tumor size, differentiation, and TNM stage, indicating a poor prognosis [39]. Although THCAT158 has not been frequently reported to be associated with tumors, it warrants further investigation. A study indicated that the lncRNA MIR205HG promoted the proliferation, migration, and invasion ability of Hepatoblastoma (HB) [40]. Taken together, these findings suggest that the candidate HRLs are involved in the progression of multiple human tumors. Therefore, it is necessary to construct a risk model based on HRLs to stratify CM patients by risk and predict their prognosis.

There is mounting evidence to suggest that hypoxia promotes tumor growth by suppressing the immune response [41, 42]. The potential involvement of long non-coding RNAs (lncRNAs) in immune regulation cannot be overlooked in hypoxic conditions. In our study, we observed a marked down-regulation of immune-related signaling pathways in high-risk CM patients. Enrichment analysis revealed that immune-related processes may mediate the role of hypoxia-related lncRNAs (HRLs) in CM patients. Furthermore, our immune function analysis demonstrated that Cluster B patients exhibited higher immune function scores compared with Cluster A patients, leading to differences in tumor growth, progression, infiltration, and angiogenesis across risk groups, ultimately resulting in poor prognosis. In summary, the differences in immune function suggest that the HRL signature we established may also reflect the infiltration of immune cells in CM and provide valuable information for clinical immunotherapy. Therefore, the HRL signature is not only a prognostic biomarker but also a predictor of tumor immune status, which can assist clinicians and physicians in better managing patients with CM.

Immunotherapy plays a crucial role in the treatment of various diseases, including cutaneous melanoma (CM). Emerging immunotherapeutic strategies for CM primarily focus on immune checkpoint inhibitors (ICIs), such as PD-1, PD-L1, CTLA4, and LAG3. In our study, we aimed to predict the response of patients with different risk levels to immunotherapy. The findings showed that patients in the high-risk group demonstrated a better response to immunotherapy. Hypoxic TME can directly affect the biological characteristics of invasive immune cells and their response to therapy. Evidence has been provided for possible mechanisms by which the hypoxic state promotes immune escape [43]. Hypoxia allows myeloid-derived suppressor cells (MDSCs) to function and have immunosuppressive activity, allowing cancer cells to evade immune surveillance and resist immune checkpoint blockades [44, 45]. In addition, as a major transcriptional regulator of tumor cell response to hypoxia, HIF-1α can interact with histone deacetylase 1 (HDAC1) and cause immune dysfunction [43, 46]. HIF-1α is also involved in the transformation of tumor-associated macrophages (tam) from antitumor phenotype (M1) to tumor-causing phenotype (M2) [47, 48]. The process of differentiation and functional implementation of T cell can also be inhibited by hypoxia through HIF-1α expression adjustment [49]. The above evidence shows the correlation between hypoxia and immune dysfunction and immune escape. We observed that patients in the high-risk group responded well to immunotherapy. Referring to the strong immunogenicity of CM, a possible explanation is that patients in the high-risk group have a strong dependence on the immune dysfunction and immune escape caused by hypoxia in the process of tumor development, so that the immune function recovery induced by immunotherapy can quickly kill tumor cells. The IPS results suggested that patients in the low-risk group benefited from anti-CTLA4, anti-PD-1, and anti-CTLA4/PD-1 immunotherapy, exhibiting higher expression of LAG3, CTLA4, PD−1, PDCD1LG2, and PD−L1. Lymphocyte-activating gene-3 (LAG-3), as a next-generation immune checkpoint, plays a critical role in regulating immune homeostasis by negatively regulating T cell activation and function [50]. CTLA4, on the other hand, is a potent inhibitor of T-cell proliferation, which prevents overactivation of the immune response [51].

Several studies have suggested that the programmed cell death-1 (PD-1)/programmed cell death ligand 1 (PD-L1) pathway plays a vital role in regulating immune responses. Targeting this pathway has been considered a breakthrough in the treatment of CM [52, 53]. Additionally, patients at different risk levels may have varying susceptibilities to anticancer drugs. High-risk group patients have been found to exhibit higher IC50 levels to pathway inhibitors such as AKT inhibitors, JNK inhibitors, and some drugs approved for anti-tumor treatment, such as Pazopanib, Lapatinib, Crizotinib, among others. These findings suggest that CM patients in different risk subgroups exhibit a promising response to antineoplastic drugs, highlighting the critical need for precisely targeted drugs and chemotherapy for CM.

## CONCLUSIONS

In this study, we developed a novel risk model based on HRLs to classify CM patients into low- and high-risk groups, which demonstrated robust sensitivity and specificity as a prognostic predictor for CM. Our functional and immune cell infiltration analyses further validated the association of this risk model with immune response. Overall, our study offers unique insights into individualized immunotherapy treatment for CM.

## Supplementary Material

Supplementary Figure 1

Supplementary Table 1

Supplementary Table 2

Supplementary Table 3
