# Supplementary material for: The development and experimental validation of hypoxia-related long noncoding RNAs prognostic signature in predicting prognosis and immunotherapy of cutaneous melanoma
Source: Aging (Albany NY). 2023 Nov 2;15(21):11918–39. doi: 10.18632/aging.205157 (PMC10683585; doi:10.18632/aging.205157)
Supplement: Supplementary Figure 1 [file aging-15-205157-s001.pdf]

SUPPLEMENTARY FIGURE

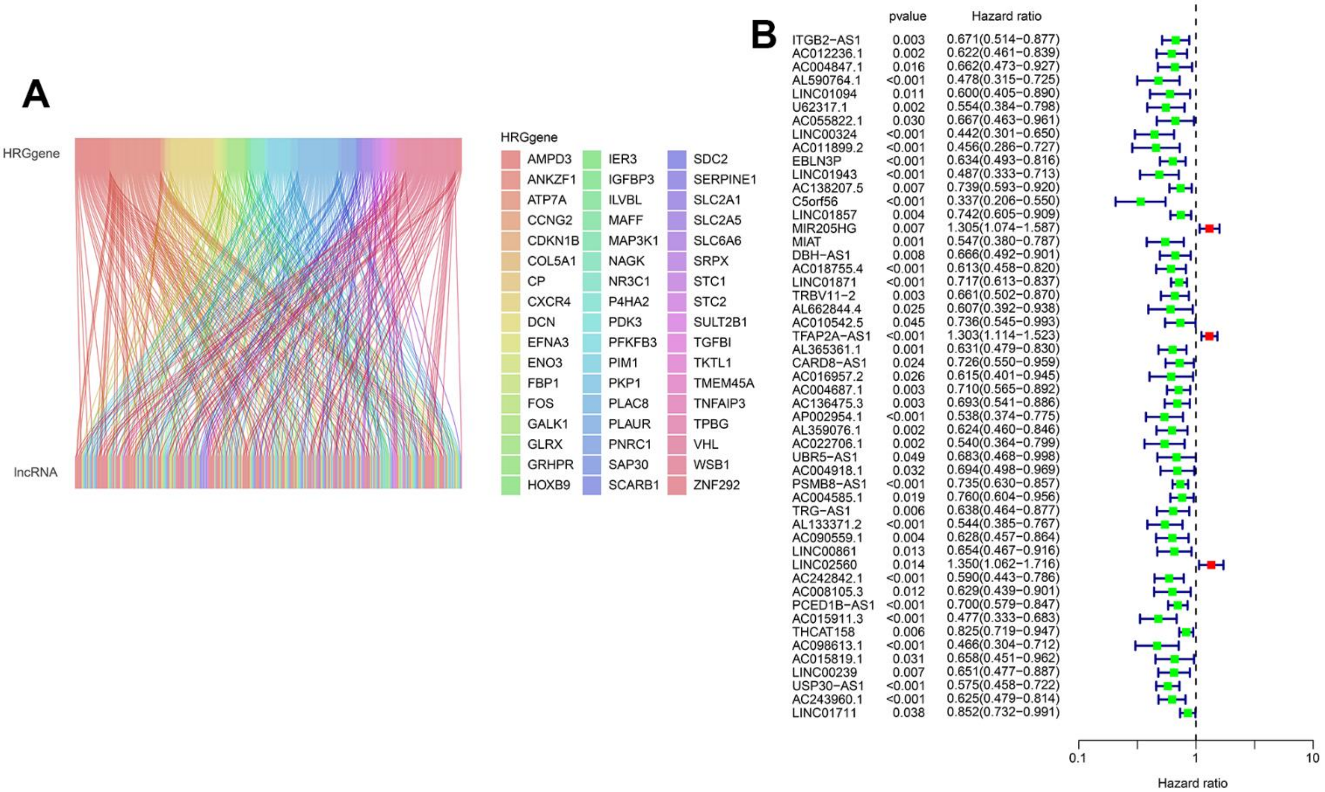

Supplementary Figure 1. Identification of prognostic HRLs for CM. (**A**) Identification of HRLs. (**B**) Univariate Cox analysis of HRLs for CM.
