# Supplementary material for: The development and experimental validation of hypoxia-related long noncoding RNAs prognostic signature in predicting prognosis and immunotherapy of cutaneous melanoma
Source: Aging (Albany NY). 2023 Nov 2;15(21):11918–39. doi: 10.18632/aging.205157 (PMC10683585; doi:10.18632/aging.205157)
Supplement: Supplementary Table 3 [file aging-15-205157-s004.pdf]

Supplementary Table 3. The gene list of prognostic related HRLs for CM.

id	HR	HR.95L	HR.95H	pvalue
ITGB2-AS1	0.671137140464625	0.513574621252859	0.877039173415981	0.00348900951483177
AC012236.1	0.621561580041301	0.460708278308383	0.838575766864853	0.00185714885662621
AC004847.1	0.662287419087852	0.473141617674038	0.92704722877333	0.0163309211976843
AL590764.1	0.478280985133198	0.315412163019371	0.7252500935607	0.000516003678495533
LINC01094	0.600491868719888	0.405236014858999	0.889828325165448	0.011031853462136
U62317.1	0.553860169394149	0.384226457710112	0.798386162862209	0.00154133262224601
AC055822.1	0.667031249640888	0.462789518326508	0.961410469291519	0.0299343132308147
LINC00324	0.442066737936786	0.300795679698848	0.649686860481925	0.0000324883045091025
AC011899.2	0.455762248212926	0.285616293583148	0.727266726593907	0.000982103145414571
EBLN3P	0.634490589736191	0.493168859030723	0.816309264244723	0.000402085735652504
LINC01943	0.487285682248631	0.332817139628501	0.713446838674111	0.000219248905195166
AC138207.5	0.738604530517063	0.593008281862057	0.919947780134428	0.00683415200776554
C5orf56	0.336688023105576	0.206142605416507	0.549904881010416	0.000013670350622382
LINC01857	0.742018308421827	0.605420167118429	0.909436454113837	0.00404691568564592
MIR205HG	1.30540847451519	1.0739512719983	1.58674916615657	0.00744119756070963
MIAT	0.547092787681263	0.380303775215042	0.787030100249755	0.00115103759627039
DBH-AS1	0.666095220817949	0.492272085045144	0.90129596350324	0.00845060687864858
AC018755.4	0.61289198876965	0.458022849572396	0.82012631083516	0.000986632410538008
LINC01871	0.716674355992356	0.613404340402243	0.837330450254474	0.0000271330719455452
TRBV11-2	0.660828968423209	0.5020173421791	0.869880159142963	0.0031369323223515
AL662844.4	0.606510655416521	0.392130500569982	0.938093758580576	0.0246300771922071
AC010542.5	0.735848918132354	0.545106252832363	0.99333593680694	0.0451085274951982
TFAP2A-AS1	1.30264568131898	1.11421479512221	1.52294313312621	0.000911217800086538
AL365361.1	0.630505868341994	0.478687184726926	0.83047481256569	0.00103219257377634
CARD8-AS1	0.726393086145709	0.5499712605248	0.959408160886062	0.0243298766170962
AC016957.2	0.615179444574919	0.400571364043521	0.944764860891026	0.0264500470048086
AC004687.1	0.70966160562014	0.564858576033377	0.891585284989277	0.0032242365942918
AC136475.3	0.6925935812453	0.541465776739557	0.885902469534869	0.0034495169559226
AP002954.1	0.538303946502239	0.373725163621561	0.775358918869353	0.000879271794920248
AL359076.1	0.624213918049454	0.460422331989397	0.846273059352005	0.0024064630659777
AC022706.1	0.539641405254255	0.364428479141049	0.799094645268039	0.00207221939928619
UBR5-AS1	0.683390210799788	0.468035469825118	0.997835015349333	0.0487025705539239
AC004918.1	0.694363600716074	0.497821932088729	0.96850053989477	0.0316752263018593
PSMB8-AS1	0.73508994231721	0.63018619297253	0.857456461791243	0.0000894811274321151
AC004585.1	0.7595671587066	0.603620564909099	0.955802870421588	0.0190015574082953
TRG-AS1	0.637950864392539	0.463991469927423	0.877131007263663	0.00565786553116376
AL133371.2	0.543516552700713	0.384993343493816	0.767312599170723	0.000529487227457248
AC090559.1	0.628246825066334	0.457011866855783	0.863640753841694	0.00419816885495861
LINC00861	0.654209349170864	0.467383602976832	0.915714350731685	0.0133923767610554
LINC02560	1.34969854740399	1.06152276516128	1.71610654867837	0.014397795867481
AC242842.1	0.590066991169284	0.443020760890873	0.78592040103813	0.000309415122333841
AC008105.3	0.629036739031298	0.439050643172713	0.901233662230337	0.0115108799395445
PCED1B-AS1	0.70001944776536	0.578559643564831	0.846977891908232	0.000244366116422486
AC015911.3	0.477123009615193	0.333467722195641	0.682663871649632	0.0000515169054696285

THCAT158	0.825451467984621	0.719369436125531	0.947176918813473	0.0062717765541732
AC098613.1	0.465653646578532	0.304498458558468	0.712099889104198	0.000420891226271034
AC015819.1	0.658427754108841	0.450677607142769	0.961945081161922	0.0307301477891111
LINC00239	0.650766834007573	0.477411523910338	0.887070066460684	0.00656481822821938
USP30-AS1	0.575066411137741	0.457935399701657	0.72215726810876	1.92486188700598E-06
AC243960.1	0.624651871984579	0.479251440969056	0.814165441808304	0.000500075355889725
LINC01711	0.851963918614458	0.73244369507368	0.990987462248395	0.0377689440282985
